# Association of Body Weight With Response to Vitamin D Supplementation and Metabolism

**DOI:** 10.1001/jamanetworkopen.2022.50681

**Published:** 2023-01-17

**Authors:** Deirdre K. Tobias, Heike Luttmann-Gibson, Samia Mora, Jacqueline Danik, Vadim Bubes, Trisha Copeland, Meryl S. LeBoff, Nancy R. Cook, I-Min Lee, Julie E. Buring, JoAnn E. Manson

**Affiliations:** 1 Division of Preventive Medicine, Department of Medicine, Brigham and Women’s Hospital and Harvard Medical School, Boston, Massachusetts; 2 Department of Nutrition, Harvard T. H. Chan School of Public Health, Boston, Massachusetts; 3 Department of Environmental Health, Harvard T. H. Chan School of Public Health, Boston, Massachusetts; 4 Division of Cardiovascular Medicine, Department of Medicine, Brigham and Women’s Hospital and Harvard Medical School, Boston, Massachusetts; 5 Cardiology Division, Massachusetts General Hospital and Harvard Medical School, Boston; 6 Division of Endocrinology, Diabetes and Hypertension, Department of Medicine, Brigham and Women’s Hospital and Harvard Medical School, Boston, Massachusetts; 7 Department of Epidemiology, Harvard T. H. Chan School of Public Health, Boston, Massachusetts

## Abstract

**Importance:**

In the Vitamin D and Omega-3 Trial (VITAL), the effects of randomized vitamin D supplementation (cholecalciferol), 2000 IU/d, reduced the risk of several health outcomes among participants with normal, but not elevated, body weights. It was unclear whether weight had any association with the outcomes of the supplementation.

**Objective:**

To investigate whether baseline body mass index (BMI) modifies vitamin D metabolism and response to supplementation.

**Design, Setting, and Participants:**

VITAL is a completed randomized, double-blind, placebo-controlled trial for the primary prevention of cancer and cardiovascular disease. In the present cohort study, an analysis was conducted in a subset of VITAL participants who provided a blood sample at baseline and a subset with a repeated sample at 2 years’ follow-up. VITAL was conducted from July 1, 2010, to November 10, 2018; data analysis for the present study was conducted from August 1, 2021, to November 9, 2021.

**Interventions:**

Treatment outcomes of vitamin D, 2000 IU/d, supplementation vs placebo associated with clinical and novel vitamin D–related biomarkers by BMI category adjusted for other factors associated with vitamin D status.

**Main Outcomes and Measures:**

Multivariable-adjusted means (SE) or 95% CIs of vitamin D–related serum biomarkers at baseline and follow-up: total 25-hydroxyvitamin D (25-OHD), 25-OHD3, free vitamin D (FVD), bioavailable vitamin D (BioD), vitamin D–binding protein (VDBP), albumin, parathyroid hormone (PTH), and calcium, and log-transformed as needed.

**Results:**

A total of 16 515 participants (mean [SD] age, 67.7 [7.0] years; 8371 women [50.7%]; 12420 non-Hispanic White [76.9%]) were analyzed at baseline, including 2742 with a follow-up blood sample. Before randomization, serum total 25-OHD levels were incrementally lower at higher BMI categories (adjusted mean [SE]: underweight, 32.3 [0.7] ng/mL; normal weight, 32.3 [0.1] ng/mL; overweight, 30.5 [0.1] ng/mL; obesity class I, 29.0 [0.2] ng/mL; and obesity class II, 28.0 [0.2] ng/mL; *P* < .001 for linear trend). Similarly, baseline 25-OHD3, FVD, BioD, VDBP, albumin, and calcium levels were lower with higher BMI, while PTH level was higher (all *P* < .001 for linear trend). Compared with placebo, randomization to vitamin D supplementation was associated with an increase in total 25-OHD, 25-OHD3, FVD, and BioD levels compared with placebo at 2 years’ follow-up, but increases were significantly lower at higher BMI categories (all treatment effect interactions *P* < .001). Supplementation did not substantially change VDBP, albumin, PTH, or calcium levels.

**Conclusions and Relevance:**

In this randomized cohort study, vitamin D supplementation increased serum vitamin D–related biomarkers, with a blunted response observed for participants with overweight or obesity at baseline. These longitudinal findings suggest that BMI may be associated with modified response to vitamin D supplementation and may in part explain the observed diminished outcomes of supplementation for various health outcomes among individuals with higher BMI.

## Key Points

**Question:**

Does body weight modify metabolism and response to vitamin D supplementation?

**Findings:**

In this cohort study, a subset including 16515 participants of the VITAL randomized clinical trial, established and novel vitamin D serum metabolite levels were on average lower at higher body mass index. Supplementation increased vitamin D levels less over 2 years at higher body mass index.

**Meaning:**

Previous trials observed reduced efficacy of vitamin D supplementation for outcomes of cancer, diabetes, and others, in subsets of participants with higher body mass index; the findings of this cohort study suggest this may be due to a blunted metabolism and internal dose at higher body weights.

## Teaser

This cohort study examines the metabolism of and response to vitamin D supplementation in individuals by levels of vitamin D–related serum biomarkers in association with adiposity.

## Introduction

Vitamin D is an essential micronutrient relevant to numerous biological processes. It is produced endogenously as vitamin D_3_ (cholecalciferol) or obtained from diet or supplements as either vitamin D_3_ or vitamin D_2_ (ergocalciferol). While the importance of vitamin D adequacy for preventing rickets and osteomalacia is established, accumulating epidemiologic evidence suggests 25-hydroxyvitamin D (25-OHD) levels may also be relevant for the incidence and progression of cancer^1^ and cardiovascular disease.^2^ In contrast, meta-analyses of randomized clinical trials of vitamin D supplementation, including the large-scale Vitamin D and Omega-3 Trial (VITAL), have not reported benefits on the primary end points of cancer or major cardiovascular disease events.^3,4,5^ However, prespecified secondary analyses in VITAL indicated a statistical interaction by baseline body weight, whereby randomization to vitamin D supplementation vs placebo was associated with a significant 24% lower cancer incidence,^3^ 42% lower cancer mortality,^6^ and 22% lower incidence of autoimmune disease^7^ among participants with normal body weight (body mass index [BMI] <25.0 [calculated as weight in kilograms divided by height in meters squared]), but no reductions among those with overweight or obesity. Moreover, 2 meta-analyses of randomized clinical trials of vitamin D supplementation and risk of type 2 diabetes similarly indicated a modifying association of BMI.^8,9^

Excess body weight and obesity are consistently characterized by lower 25-OHD blood levels and a higher prevalence of vitamin D insufficiency and deficiency in cross-sectional studies.^10^ Evidence from a bidirectional mendelian randomization study implicated obesity as a driver of low serum 25-OHD levels, rather than the reverse, although reasons for this observation are largely unknown.^11^ Furthermore, vitamin D supplementation has not been shown to modify body weight^12^ or body composition in VITAL participants,^13^ indicating weight gain precedes alterations in vitamin D metabolism rather than vice versa. In VITAL, mean serum total 25-OHD levels with active supplementation at 1 year follow-up were significantly lower for participants with obesity vs normal weight (38.6 vs 45.9 ng/mL [to convert to nanomoles per liter, multiply by 2.496]), despite receiving the same dose.^3^ Hypotheses for diminished response to vitamin D supplementation in obesity include that a greater amount of vitamin D, a fat-soluble vitamin, is sequestered in adipose tissue, leaving lower amounts in circulation.^14^ Volumetric dilution of vitamin D across a larger adiposity mass has also been posited.^15^ Alternatively, obesity may suppress hepatic enzyme 25-hydroxylation of vitamin D to 25-OHD, thereby reducing bioactivity.^16^ It remains unknown whether the blunted response of 25-OHD levels with supplementation at higher body weights also extends to other biomarkers of vitamin D metabolism. If so, diminished vitamin D bioavailability or activity would be plausible explanations for the limited effectiveness of vitamin D supplementation to reduce cancer incidence and mortality, type 2 diabetes,^17^ and other pertinent outcomes at higher BMIs.

We hypothesized that, in addition to differences in circulating total 25-OHD levels with vitamin D supplementation, obesity would modify other circulating markers of vitamin D bioavailability and activity. Of total vitamin D in circulation, 85% to 90% is bound to a vitamin D–binding protein (VDBP). Bioavailable 25-OHD (BioD) is the remaining 10% to 15% vitamin D fraction in circulation that is not bound to VDBP; BioD is primarily bound to albumin. Free vitamin D (FVD) levels represent less than 1% of total vitamin D. Identifying subgroups with suboptimal response to supplementation underscores the potential role for future precision prevention strategies to tailor dosing and maximize the effectiveness of vitamin D supplementation for chronic disease prevention. We investigated this aim in a post hoc analysis of the randomized VITAL, with repeated measures at baseline and 2 years for total vitamin D, FVD, BioD, VDBP, albumin, parathyroid hormone (PTH), and calcium.

## Methods

### Study Population

**Figure 1.**
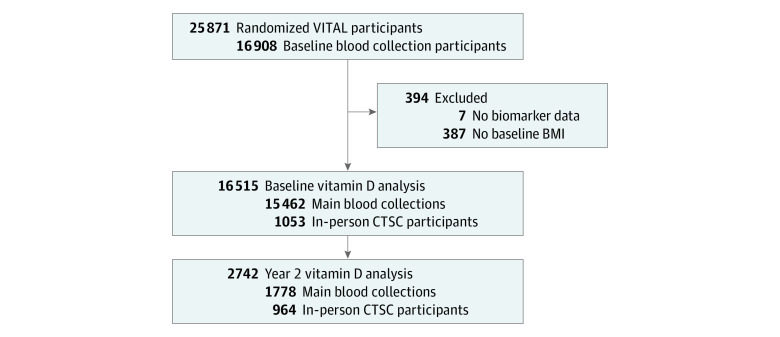
Flowchart Diagram of Eligible Vitamin D and Omega-3 Trial (VITAL) Participants Included in Analyses BMI indicates body mass index; CTSC, Clinical and Translational Science Center.

We performed a post hoc analysis in VITAL, a completed randomized, double-blind, placebo-controlled 2 × 2 factorial trial of vitamin D_3_ (cholecalciferol), 2000 IU/d, and marine ω-3 fatty acids, 1 g/d, for the primary prevention of cancer and cardiovascular disease (NCT01169259).^3^ VITAL was conducted from July 1, 2010, to November 10, 2018; data analysis for the present study was conducted from August 1, 2021, to November 9, 2021. Eligible participants were men aged 50 years or older and women aged 55 years or older who were free of cancer and cardiovascular disease at baseline enrollment. Details of the trial population, results, and blood collection methods were previously described in detail.^18,19^ Briefly, after a 3-month placebo run-in period to demonstrate adherence, participants were randomized in 5-year age groups, using a computer-generated table of random numbers, to 1 of the 4 treatment combinations. Investigators and participants were blinded to active vs placebo study pills. Among the 25 871 individuals randomized, there were 16 515 eligible participants who contributed baseline blood samples before randomization (October 2010 to March 2014) and, among them, 2742 provided a blood sample at 2 years’ follow-up (Figure 1). These samples were collected via mail-based kits, in-home visits, or phlebotomy centers and shipped overnight to the Boston laboratory where they were processed and stored at −170 °C within 36 hours of the sample draw. Height and body weight were self-reported on the baseline questionnaire. In-person visits were conducted for 1054 Boston-area participants who attended the Brigham and Women’s Hospital in Boston for detailed phenotyping and biospecimen collections at randomization and again at 2 years’ follow-up. If participants provided blood samples for both the in-person and mail-based collections, we analyzed the in-person values. Participants provided written informed consent at enrollment and the study protocol was approved by the Partners Institutional Review Board, Boston, Massachusetts. Participants received financial compensation as part of the original trial. This study followed the Strengthening the Reporting of Observational Studies in Epidemiology (STROBE) reporting guideline for observational studies.

### Biomarker Assays and Quality Control

Baseline and follow-up samples were shipped and assayed in tandem to avoid systematic error, and personnel were blinded to collection and randomized treatment status. Total 25-OHD and 25-OHD3 levels were quantified with liquid chromatography tandem mass spectrometry (Quest Diagnostics Nichols Institute).^13^ Free vitamin D levels were measured using an enzyme-linked immunoassay and polyclonal VDBD (Future Diagnostics Solutions BV). We derived BioD, defined as circulating 25-OHD not bound to VDBP, with the validated equation by Powe et al^20^ (eMethods in Supplement 1). Albumin levels (Beckman Coulter Clinical Chemistry AU analyzer; Quest Diagnostics Nichols Institute) and intact PTH, using a chemiluminescent-based assay (Beckman Coulter), were measured, and serum calcium levels were quantified via spectroscopy (Quest Diagnostics Nichols Institute). Coefficients of variation were calculated from blinded duplicate aliquots of 20 participant samples: total 25-OHD = 4.9%, 25-OHD3 = 4.1%, FVD = 6.8%, VDBP = 15.7%, PTH = 7.6%, and calcium = 2.0%. VITAL participated in the vitamin D standardization program of the Centers for Disease Control and Prevention.^21^

### Statistical Analysis

Our analyses excluded participants with missing or extreme baseline BMI (BMI<12.0 or ≥60.0). Participants’ baseline characteristics, demographic characteristics, and health status at trial baseline were stratified by baseline BMI categories of underweight (<18.5), normal weight (18.5-24.9), overweight (25.0-29.9), obesity class I (30.0-34.9), and obesity class II (≥35.0). For analyses including repeated biomarkers at 2 years, we combined the underweight and normal weight categories due to an insufficient sample size for BMI less than 18.5.

To minimize outliers, we winsorized biomarker values at the bottom and top 0.5%, then inspected the biomarker distributions for normality. BioD, VDBP, PTH, and calcium were log transformed for analysis to improve normal distribution. We performed multivariable-adjusted generalized linear regression models with biomarkers as the continuous dependent variable to calculate the least square mean (SEM) or geometric mean and 95% CI for log-transformed biomarkers, stratified by baseline BMI category. We modeled continuous BMI to calculate the *P* value linear trends. Multivariable models were adjusted for baseline characteristics of age in years (continuous), sex, prevalent diabetes, pretrial use of vitamin D supplements, smoking status (current, past, never; not reported [1.3%]), leisure-time physical activity (above vs below median = 16.6 metabolic equivalent task hours/week; not reported [<1.0%]), self-reported race and ethnicity, as required by the funding agency and categorized in the electronic health records (Asian or Pacific Islander, Black, Native American or Alaska Native, non-Black Hispanic, non-Hispanic White, other/unknown (option indicated by participant with no further breakdown available); not reported [2.2%]), US geographic region (West, South, Midwest, Northeast), season at blood draw (winter, spring, summer, fall), and Brigham and Women’s Hospital Clinical and Translational Science Center subcohort participation. Categorical covariates were modeled with indicator variables for not reported covariate data, as the amount of not reported or missing data was small.

We generated waterfall plots to visualize the variability among participants for changes in total serum 25-OHD concentration at 2 years. Means and SEM (or geometric means and 95% CI for log-transformed biomarkers) of the continuous biomarkers were estimated from multivariable linear mixed-effects models with maximum likelihood estimation of the covariance parameters with an unstructured covariance matrix. The treatment effects per BMI category were calculated as the mean change difference in biomarkers, plus additional adjustment for the baseline biomarker value in an analysis of covariance.^22,23^ We tested for heterogeneity via a 2-way multiplicative interaction term of randomized treatment assignment × BMI added to the main effects model.

In secondary analyses, we estimated the treatment effect at 2 years according to categories of central obesity restricted to the subset of participants with in-person technician measurements. Classification of central obesity was defined based on the World Health Organization waist circumference (WC) for minimal risk (category I) including women less than 80.0 cm and men less than 94.0; moderate risk (category II) including women 80.0 to 87.9 cm and men 94.0 to 101.9 cm; and high risk (category III) including women, 88.0 cm or more and men, 102.0 cm or more. We also performed a sensitivity analysis among participants with baseline total 25-OHD levels less than 20.0 ng/mL to estimate whether effect modification by BMI persisted among those with initially insufficient vitamin D levels. We used SAS, version 9.4 (TS1M5) statistical software (SAS Institute Inc) to perform the analysis, with an a priori level of significance set as *P* < .05 with 2-sided hypothesis tests.

## Results

Of the 16 908 VITAL participants in the baseline blood collection, 16 515 had vitamin D–related biomarkers assayed and were eligible for analysis, with mean (SD) age 67.7 (7.0) years (Figure 1). There were 238 (1.5%) who self-reported Asian or Pacific Islander race andethnicity, 2445 (15.1%) as Black, 129 (0.8%) Native American or Alaska Native, 589 (3.6%) non-Black Hispanic, 12420 (76.9%) non-Hispanic White, and 333 (2.1%) identifying as other or unknown (with no available further breakdown of category). Baseline characteristics according to BMI category are summarized in Table 1. A total of 8371 women (50.7%) and 8144 men (49.3%) were included in the analysis. Participants with overweight (6688 [40.5%]) or obesity (4457 [27.0%]) were, on average, younger and more likely to self-report Black race and ethnicity, a lower household annual income, and a lower achieved educational level than participants with normal body weight. Participants who were obese were less likely to be physically active or report alcohol intake, but smoking status was similar across BMI categories. We observed similar findings by BMI category for the subset of participants included in the 2-year change analyses (eTable 1 in Supplement 1).

**Table 1.** Characteristics of 16 515 VITAL Participants Included in Biomarker Analysis by BMI at Study Baseline

Characteristic	Baseline randomization, No. (%)				
	Underweight, BMI <18.5	Normal, BMI 18.5-24.9	Overweight, BMI 25.0-29.9	Obesity I, BMI 30.0-34.9	Obesity II, BMI ≥35.0
No.	149	5221	6688	2851	1606
BMI, mean (SD)	17.4 (1.2)	22.8 (1.6)	27.3 (1.4)	32.1 (1.4)	39.8 (4.6)
Age, mean (SD), y	70.6 (7.0)	68.9 (7.1)	67.8 (6.9)	66.7 (6.6)	65.4 (6.3)
Sex					
Male	33 (22.1)	2294 (43.9)	3896 (58.3)	1374 (48.2)	547 (34.1)
Female	116 (77.9)	2927 (56.1)	2792 (41.7)	1477 (51.8)	1059 (65.9)
Race and ethnicity					
Asian or Pacific Islander	4 (2.8)	141 (2.8)	79 (1.2)	9 (0.3)	5 (0.3)
Black	11 (7.6)	420 (8.2)	892 (13.6)	605 (21.7)	517 (33.0)
Native American or Alaska Native	0	29 (0.6)	47 (0.7)	35 (1.3)	18 (1.1)
Non-Black Hispanic	3 (2.1)	141 (2.8)	262 (4.0)	115 (4.1)	68 (4.3)
Non-Hispanic White	122 (84.7)	4279 (83.6)	5124 (78.4)	1959 (70.3)	936 (59.7)
Other or unknown^a^	4 (2.8)	106 (2.1)	135 (2.1)	64 (2.3)	24 (1.5)
Highest educational level					
No high school	3 (2.0)	21 (0.4)	57 (0.9)	26 (0.9)	52 (3.2)
High school	10 (6.8)	333 (6.4)	591 (8.8)	351 (12.4)	248 (15.5)
College	57 (38.8)	2001 (38.4)	2734 (40.9)	1327 (46.7)	742 (46.3)
Postcollege	77 (52.4)	2859 (54.8)	3300 (49.4)	1139 (40.1)	560 (35.0)
Exercise, mean (SD), MET-h/wk	27.4 (28.9)	28.8 (26.8)	23.8 (24.2)	17.9 (21.8)	12.4 (22.5)
Smoking					
Never	80 (54.4)	2822 (54.7)	3310 (50.2)	1407 (50.0)	824 (52.2)
Past	47 (32.0)	2016 (39.1)	2928 (44.4)	1254 (44.5)	642 (40.7)
Current	20 (13.6)	324 (6.3)	359 (5.4)	155 (5.5)	113 (7.2)
Alcohol intake					
Never	37 (25.2)	1280 (24.9)	1715 (26.0)	1020 (36.4)	769 (48.7)
<1/wk	10 (6.8)	314 (6.1)	451 (6.8)	258 (9.2)	144 (9.1)
1-6/wk	64 (43.5)	1873 (36.4)	2422 (36.7)	950 (33.9)	481 (30.5)
Daily	36 (24.5)	1681 (32.7)	2012 (30.5)	575 (20.5)	185 (11.7)
Diabetes	10 (6.7)	305 (5.8)	774 (11.6)	621 (21.8)	560 (34.9)
Hypertension medication	39 (26.2)	1865 (35.7)	3368 (50.4)	1815 (63.6)	1237 (77.0)
Cholesterol-lowering medication	29 (19.5)	1521 (29.1)	2799 (41.9)	1278 (44.8)	761 (47.4)
Pretrial supplemental vitamin D	57 (38.3)	2472 (47.3)	3655 (54.7)	1682 (59.0)	1016 (63.3)
Geographic region					
West	43 (28.9)	1065 (20.4)	1174 (17.6)	426 (14.9)	182 (11.3)
South	43 (28.9)	1789 (34.3)	2461 (36.8)	1102 (38.7)	662 (41.2)
Midwest	27 (18.1)	1040 (19.9)	1385 (20.7)	686 (24.1)	438 (27.3)
Northeast	36 (24.2)	1327 (25.4)	1668 (24.9)	637 (22.3)	324 (20.2)
Season at blood draw					
Winter	22 (14.8)	928 (17.8)	1296 (19.4)	603 (21.1)	377 (23.5)
Spring	22 (14.8)	861 (16.5)	1127 (16.9)	541 (19.0)	332 (20.7)
Summer	47 (31.5)	1417 (27.1)	1745 (26.1)	717 (25.2)	412 (25.7)
Fall	58 (38.9)	2015 (38.6)	2520 (37.7)	990 (34.7)	485 (30.2)
Provided 2-y repeated blood sample	26 (17.4)	724 (13.9)	1072 (16.0)	547 (19.2)	373 (23.2)
CTSC substudy participation	12 (8.1)	359 (6.9)	447 (6.7)	160 (5.6)	75 (4.7)

Abbreviations: BMI, body mass index (calculated as weight in kilograms divided by height in meters squared); CTSC, Clinical and Translational Science Center; MET, metabolic equivalent of tasks; VITAL, Vitamin D and Omega-3 Trial.

^a^ Defined as the option indicated by participant with no further breakdown available.

Mean (SD) serum 25-OHD level before the use of study pills was 30.6 (9.5) ng/mL and most levels (14 279 [87.5%]) were sufficient at 20.0 ng/mL or greater. Total 25-OHD levels were significantly lower among participants at higher BMI categories, even with adjustment for other factors associated with serum vitamin D levels (Table 2); mean (SE) for underweight (32.3 [0.7] ng/mL), normal weight (32.3 [0.1] ng/mL), overweight (30.5 [0.1] ng/mL), obesity class I (29.0 [0.2] ng/mL), and obesity class II (28.0 [0.2] ng/mL) (*P* < .001). Statistically significant inverse trends were also observed with lower baseline levels of 25-OHD3, FVD, BioD, VDBP, albumin, and calcium at higher BMI, and higher PTH levels with higher BMI.

**Table 2.** Multivariable-Adjusted Mean or Geometric Mean Vitamin D–Related Biomarker Concentrations at Baseline, by BMI^a^

Characteristic	No.	BMI at baseline randomization, multivariable-adjusted mean (SEM) [N = 16 516]					*P* value for continuous BMI
		<18.5	18.5-24.9	25.0-29.9	30.0-34.9	≥35.0	
Total 25-OHD, ng/mL	16 375	32.6 (0.7)	32.4 (0.1)	30.6 (0.1)	29.0 (0.2)	28.0 (0.2)	<.001
25-OHD3, ng/mL	16 369	31.8 (0.7)	31.9 (0.1)	30.1 (0.1)	28.5 (0.2)	27.2 (0.2)	<.001
Free vitamin D, pg/mL	5175	6.48 (0.27)	6.39 (0.04)	5.93 (0.04)	5.65 (0.06)	5.45 (0.09)	<.001
Bioavailable D, geometric mean (95% CI), ng/mL	5168	2.3 (2.1-2.5)	2.4 (2.3-2.4)	2.2 (2.2-2.2)	2.1 (2.0-2.1)	1.9 (1.9-2.0)	<.001
VDBP, mg/mL	5175	467 (442-494)	472 (467-476)	462 (459-466)	452 (446-458)	455 (447-464)	<.001
Albumin, g/dL	16 470	4.43 (0.02)	4.37 (0.00)	4.35 (0.00)	4.31 (0.01)	4.22 (0.01)	<.001
PTH, geometric mean (95% CI), pg/mL	16 419	33.5 (31.3-35.8)	33.3 (32.9-33.7)	35.7 (35.4-36.1)	37.8 (37.3-38.4)	40.3 (39.4-41.1)	<.001
Calcium, geometric mean (95% CI), mg/dL	15 536	9.40 (9.34-9.47)	9.40 (9.39-9.41)	9.38 (9.37-9.39)	9.35 (9.34-9.37)	9.30 (9.28-9.32)	<.001

Abbreviations: BMI, body mass index (calculated as weight in kilograms divided by height in meters squared); 25-OHD, 25-hydroxyvitamin D; PTH, parathyroid hormone; VDBP, vitamin D binding protein.

SI conversion: To convert albumin to grams per liter, multiply by 10; calcium to millimoles per liter, multiply by 0.25; 25-OHD to nanomoles per liter, multiply by 2.496; PTH to nanograms per liter, multiply by 1.

^a^ Values were adjusted for baseline factors: age (continuous), sex (male, female), prevalence of type 2 diabetes, preintervention vitamin D supplement use, smoking status (never, past, current, not reported), total physical activity (above, below median = 16.6 MET-h/wk), race and ethnicity (Asian or Pacific Islander, Black, Native American or Alaska Native, non-Black Hispanic, non-Hispanic White, other/unknown, not reported), geographic region (West, South, Midwest, Northeast), season of blood draw (winter, spring, summer, fall), and in-person Clinical and Translational Science Center subgroup participation.

**Figure 2.**
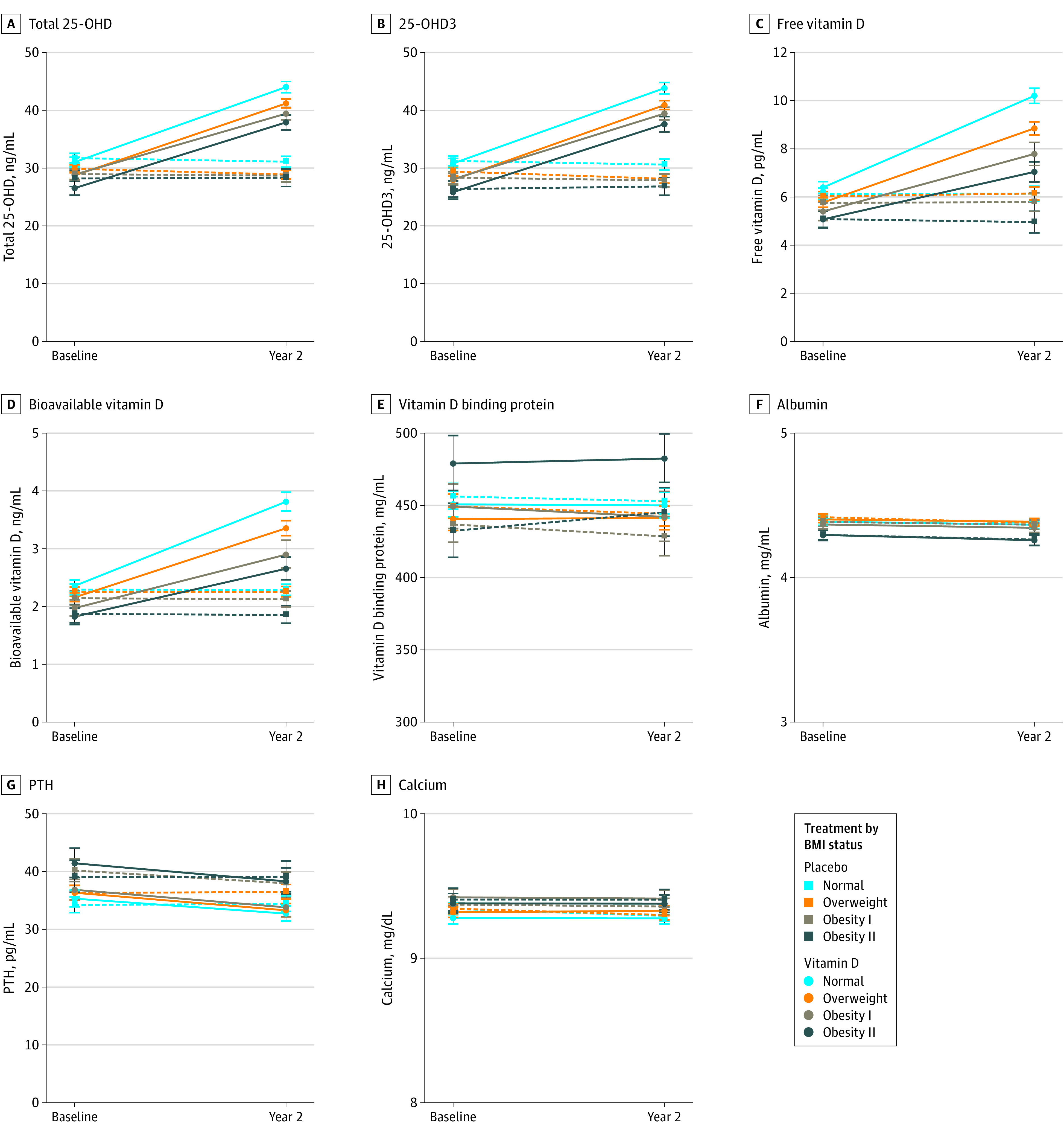
Multivariable-Adjusted Mean Vitamin D–Related Biomarker Concentrations at Baseline and 2 Years’ Follow-up, by Randomized Treatment Assignment and Baseline Body Mass Index (BMI) Concentrations shown for total 25-hydroxyvitamin D (25-OHD) (A), 25-OHD3 (B), free vitamin D (C), bioavailable vitamin D (D), vitamin D–binding protein (E), albumin (F), parathyroid hormone (PTH) (G), and calcium (H). Circles and bars are least square mean (standard error of the mean) or geometric mean (95% CI) adjusted for baseline factors: age (continuous), sex (male, female), prevalence of type 2 diabetes, preintervention vitamin D supplement use, smoking status (never, past, current, not reported), total physical activity (above, below median = 16.6 metabolic equivalents of task hours per week), race and ethnicity (Asian or Pacific Islander, Black, Native American or Alaska Native, non-Black Hispanic, non-Hispanic White, other/unknown, not reported), geographic region (West, South, Midwest, Northeast), season of blood draw (winter, spring, summer, fall), and in-person Clinical and Translational Science Center subgroup participation. BMI indicates body mass index. To convert albumin to grams per liter, multiply by 10; calcium to millimoles per liter, multiply by 0.25; 25-OHD to nanomoles per liter, multiply by 2.496; PTHe to nanograms per liter, multiply by 1.

Among the 2742 participants with repeated blood collections at year 2, there was a significant mean (SD) 11.9 (8.6) ng/mL increase in serum 25-OHD level in the group randomized to vitamin D supplementation, compared with little change in the placebo group (−0.7 [7.9] ng/mL). Waterfall plots (eFigure in Supplement 1) illustrate the variability of within-person change in total 25-OHD level according to randomized treatment. The multivariable-adjusted means at baseline and 2 years for all biomarkers by treatment group and BMI category are given in Figure 2 and Table 3. There were significant increases in the mean total 25-OHD, 25-OHD3, FVD, and BioD levels at 2 years among participants randomized to active supplementation, and little or no changes observed for the placebo group. Furthermore, we observed statistically significant interactions (all interactions *P* < .001) by baseline BMI category for these treatment effects vs placebo, whereby the magnitudes of biomarker increases were lower at higher baseline BMI. For example, for total 25-OHD level, the mean (SD) increases at 2 years for supplementation vs placebo across BMI strata were 13.5 (0.6) ng/mL for less than 25.0, 12.7 (0.5) ng/mL for 25.0 to 29.9, 10.5 (0.7) ng/mL for 30.0 to 34.9, and 10.0 (1.0) ng/mL for greater than or equal to 35.0 ng/mL. In contrast, there was no significant difference with vitamin D supplementation vs placebo in VDBP, albumin, PTH, or calcium levels, and the lack of association was observed for all BMI categories.

**Table 3.** Multivariable-Adjusted Mean or Geometric Mean Vitamin D–Related Biomarkers at Baseline and 2 Years’ Follow-up, by Randomized Treatment Assignment and BMI^a^

Biomarker, BMI category^b^	Multivariable-adjusted mean (SEM)				Treatment effect, mean (SE)	*P* value for treatment effect interaction by BMI
	Placebo		Vitamin D			
	Baseline	Year 2	Baseline	Year 2		
**Total 25-OHD, ng/mL**						
<25.0	31.7 (0.4)	31.1 (0.5)	31.0 (0.4)	44.0 (0.5)	13.5 (0.6)	<.001
25.0-29.9	29.9 (0.4)	28.9 (0.4)	28.7 (0.4)	41.2 (0.4)	12.7 (0.5)	
30.0-34.9	29.0 (0.5)	28.7 (0.6)	28.9 (0.5)	39.4 (0.5)	10.5 (0.7)	
≥35.0	28.2 (0.7)	28.3 (0.8)	26.5 (0.6)	37.9 (0.7)	10.0 (1.0)	
**25-OHD3, ng/mL**						
<25.0	31.2 (0.4)	30.6 (0.5)	30.8 (0.4)	43.8 (0.5)	13.5 (0.6)	<.001
25.0-29.9	29.4 (0.4)	28.1 (0.4)	28.0 (0.4)	40.9 (0.4)	13.4 (0.5)	
30.0-34.9	28.4 (0.5)	27.9 (0.6)	28.1 (0.5)	39.4 (0.6)	11.6 (0.7)	
≥35.0	26.4 (0.7)	26.8 (0.8)	25.8 (0.6)	37.6 (0.7)	10.5 (1.0)	
**Free vitamin D, pg/mL**						
<25.0	6.13 (0.12)	6.14 (0.16)	6.39 (0.12)	10.20 (0.16)	3.84 (0.19)	<.001
25.0-29.9	6.03 (0.10)	6.15 (0.14)	5.77 (0.10)	8.85 (0.14)	2.92 (0.17)	
30.0-34.9	5.75 (0.16)	5.79 (0.20)	5.40 (0.19)	7.79 (0.24)	2.57 (0.32)	
≥35.0	5.08 (0.18)	4.96 (0.23)	5.07 (0.17)	7.04 (0.21)	2.22 (0.34)	
**Bioavailable vitamin D, geometric mean (95% CI), ng/mL**						
<25.0	2.3 (2.2-2.4)	2.3 (2.2-2.4)	2.4 (2.3-2.5)	3.8 (3.7-4.0)	1.5 (0.1)	<.001
25.0-29.9	2.3 (2.2-2.3)	2.3 (2.2-2.3)	2.2 (2.1-2.2)	3.4 (3.2-3.5)	1.2 (0.1)	
30.0-34.9	2.1 (2.0-2.3)	2.1 (2.0-2.3)	2.0 (1.8-2.1)	2.9 (2.7-3.1)	1.0 (0.1)	
≥35.0	1.9 (1.7-2.0)	1.9 (1.7-2.0)	1.8 (1.7-2.0)	2.7 (2.5-2.9)	0.9 (0.1)	
**VDBP, geometric mean (95% CI), mg/mL**						
<25.0	456 (447-465)	453 (444-461)	451 (443-460)	450 (441-459)	1 (5)	.56
25.0-29.9	449 (441-458)	444 (436-453)	441 (433-449)	441 (433-450)	3 (4)	
30.0-34.9	437 (424-449)	429 (415-442)	449 (434-465)	442 (425-460)	0 (10)	
≥35.0	432 (414-451)	445 (429-462)	479 (460-498)	482 (466-499)	6 (13)	
**Albumin, g/dL**						
<25.0	4.40 (0.01)	4.38 (0.01)	4.39 (0.01)	4.37 (0.01)	0.00 (0.02)	.39
25.0-29.9	4.42 (0.01)	4.38 (0.01)	4.40 (0.01)	4.38 (0.01)	0.02 (0.01)	
30.0-34.9	4.39 (0.02)	4.37 (0.02)	4.37 (0.02)	4.34 (0.02)	−0.01 (0.02)	
≥35.0	4.30 (0.02)	4.26 (0.02)	4.29 (0.02)	4.26 (0.02)	0.00 (0.02)	
**PTH, geometric mean (95% CI), pg/mL**						
<25.0	34.2 (32.9-35.6)	34.4 (33.1-35.7)	35.3 (33.9-36.8)	32.7 (31.5-34.0)	−2.1 (0.9)	.47
25.0-29.9	36.3 (35.0-37.6)	36.5 (35.2-37.8)	36.3 (35.1-37.6)	33.3 (32.1-34.5)	−4.0 (0.8)	
30.0-34.9	40.2 (38.3-42.2)	38.0 (36.1-39.9)	36.8 (35.1-38.6)	33.8 (32.2-35.5)	−2.0 (1.3)	
≥35.0	39.1 (36.4-41.9)	39.1 (36.5-41.8)	41.4 (39.0-44.0)	38.3 (36.1-40.6)	−3.6 (1.8)	
**Calcium, geometric mean (95% CI), mg/dL**						
<25.0	9.34 (9.30-9.38)	9.29 (9.25-9.33)	9.28 (9.24-9.32)	9.28 (9.24-9.32)	0.01 (0.03)	.40
25.0-29.9	9.34 (9.31-9.38)	9.30 (9.27-9.33)	9.32 (9.28-9.35)	9.33 (9.29-9.36)	0.05 (0.02)	
30.0-34.9	9.37 (9.32-9.43)	9.36 (9.30-9.41)	9.42 (9.37-9.48)	9.41 (9.36-9.47)	−0.01 (0.04)	
≥35.0	9.41 (9.33-9.48)	9.41 (9.34-9.48)	9.38 (9.31-9.45)	9.38 (9.32-9.44)	−0.02 (0.05)	

Abbreviations: BMI, body mass index (calculated as weight in kilograms divided by height in meters squared); 25-OHD, 25-hydroxyvitamin D; PTH, parathyroid hormone; SEM, standard error of the mean; VDBP, vitamin D binding protein.

SI conversion: To convert albumin to grams per liter, multiply by 10; calcium to millimoles per liter, multiply by 0.25; 25-OHD to nanomoles per liter, multiply by 2.496; PTH to nanograms per liter, multiply by 1.

^a^ Values were adjusted for baseline factors: age (continuous), sex (male, female), prevalence of type 2 diabetes, preintervention vitamin D supplement use, smoking status (never, past, current, not reported), total physical activity (above, below median = 16.6 MET-h/week), race and ethnicity (Asian or Pacific Islander, Black, Native American or Alaska Native, non-Black Hispanic, non-Hispanic White, other/unknown, not reported), geographic region (West, South, Midwest, Northeast), season of blood draw (winter, spring, summer, fall), and in-person Clinical and Translational Science Center subgroup participation. Mean treatment effects were additionally adjusted for baseline biomarker concentrations. Tests for interaction of treatment effect by baseline BMI category were additionally adjusted for baseline biomarker concentrations and baseline biomarker × BMI interaction.

^b^ Sample sizes included for each biomarker: total 25-OHD, n = 2635; 25-OHD3, n = 2632; free vitamin D, n = 964; bioavailable vitamin D, n = 963; VDBP, n = 964; albumin, n = 2732; PTH, n = 2730; calcium, n = 1997.

We repeated the above models of treatment effect heterogeneity by baseline WC categories for the subset of 964 Clinical and Translational Science Center participants with in-person measurements (eTable 2 in Supplement 1). As with BMI-derived cut points, we observed significant increases in total 25-OHD, 25-OHD3, FVD, and BioD levels with supplementation vs placebo that were blunted at the higher WC-derived obesity categories. For example, vitamin D supplementation vs placebo was associated with a mean (SD) 13.1 (0.9) ng/mL greater increase in total 25-OHD level for participants in the lowest WC category, compared with 12.3 (1.0) ng/mL for the WC central obesity category II and 11.6 (0.6) ng/mL for the WC central obesity category III (*P* < .001 for interaction). Supplementation was not related to changes in VDBP, albumin, PTH, or calcium levels in this subset, nor did these associations differ by WC category.

In a sensitivity analysis, we evaluated treatment effect heterogeneity by baseline BMI, restricted to 377 (14.6%) participants with low vitamin D levels at baseline (total 25-OHD <20.0 ng/mL) (eTable 3 in Supplement 1). The overall trends in these findings were like the overall analysis; however, statistical power for specific between-group comparisons was limited.

## Discussion

Our analysis of older US adults from the randomized VITAL presents novel hypothesis-generating evidence that adiposity blunts the response to vitamin D supplementation. Consistent with prior cross-sectional evidence, higher overall and central obesity were associated with significantly lower serum total 25-OHD levels in our large sample size of 16 515 participants at baseline, controlling for numerous variables used to estimate vitamin D levels. Furthermore, treatment with vitamin D_3_, 2000 IU/d, supplementation vs placebo significantly increased total 25-OHD, 25-OHD3, FVD, and BioD levels at 2 years. However, the magnitude of response to supplementation was notably lower among those with higher BMI, including reduced increases in total 25-OHD levels, as well as novel markers of circulating vitamin D activity. Thus, increases in vitamin D availability and bioactivity achieved with supplementation may be modestly or moderately diminished with excess adiposity. Our innovative longitudinal analyses adjusted for other factors and potential confounders of vitamin D status change. These trends were also observed among the subgroup of participants with low vitamin D levels at baseline, suggesting that, even when insufficient for vitamin D, obesity may still blunt the response to supplementation. There was, however, no associations between supplemental vitamin D, overall or by BMI, and the vitamin D carrier proteins VDBP or albumin. Parathyroid hormone and calcium levels were also unaffected by supplementation vs placebo, regardless of BMI category. Collectively, this evidence warrants further investigation into impaired pharmacokinetics or efficacy of vitamin D supplementation for populations with higher BMI.

Several authors of the present study previously explored factors associated with changes in serum 25-OHD levels from baseline to 1-year follow-up in VITAL and identified minoritized race and ethnicity, baseline 25-OHD levels, and nonrandomized supplementation as factors related to the magnitude of change.^24^ This current examination of an expanded profile of vitamin D metabolites and biomarkers provides a novel snapshot of vitamin D availability and activity beyond total 25-OHD. Vitamin D status is typically evaluated with total serum 25-OHD levels, but this composite of bound and unbound 25-OHD may not accurately reflect the bioactive subfraction (FVD) and may bias estimates of vitamin D deficiency. Indeed, the main VITAL reported no significant difference in efficacy of supplementation on cancer incidence by baseline serum 25-OHD levels less than 20.0 ng/mL (hazard ratio [HR], 0.97; 95% CI, 0.68-1.39) vs greater than or equal to 20.0 ng/mL (HR, 0.98; 95% CI, 0.86-1.12). However, there was significant effect modification by baseline BMI for the association between vitamin D supplementation with cancer incidence. That is, even though most serum 25-OHD levels were sufficient, vitamin D supplementation was related to a significant reduction in cancer incidence for BMI less than 25. In contrast, despite having mean lower serum levels of 25-OHD at baseline, there was no association between vitamin D treatment and cancer incidence among participants with overweight (HR, 1.04; 95% CI, 0.90-1.21) or obesity (HR, 1.13; 95% CI, 0.94-1.37).

There are conflicting hypotheses as to why higher BMI might be associated with lower 25-OHD circulating levels or activity. One theory proposes that, as a fat-soluble vitamin, there is a greater removal of vitamin D from circulation due to increased storage capacity across higher adiposity volumes.^25,26^ Our results are largely consistent with this hypothesis. Evidence from weight loss interventions also point to vitamin D sequestration as a function of adiposity amount. For example, the amount of weight loss was associated with greater increases in serum 25-OHD levels among postmenopausal women during a randomized weight loss intervention, despite no changes in dietary or supplemental vitamin D intakes.^27^ Similarly, a cohort of patients undergoing Roux-en-Y gastric bypass bariatric surgery also had a significant increase in circulating vitamin D levels concurrent with extreme weight loss, even without receiving vitamin D supplementation.^28^ Thus, BMI-related storage capacity and sequestration may contribute to the moderate differences by BMI in circulating levels of 25-OHD noted with supplementation.

Another hypothesis posits obesity-induced hepatic dysfunction contributes to impaired vitamin D metabolism. Oral vitamin D enters circulation and is activated enzymatically in the liver to 25-OHD by cytochrome P450 (CYP) enzymes. Interference in metabolism by obesity and related cardiometabolic dysfunction would result in a dampened response to vitamin D supplementation on the amount of circulating 25-OHD and its downstream activity. Animal models demonstrated downregulation of CYP2R1 in obesity and diabetes.^29^ It was also shown that surgically induced weight loss in humans led to increased CYP2R1 activity in adipose tissue.^29^ Indeed, the effects of supplementation with vitamin D_3_, 2000 IU/d, on serum 25-OHD, FVD, and BioD levels were markedly attenuated across higher BMI categories. Prior evidence for central obesity, reflecting underlying viscerally accumulated adipose tissue and hepatic fat depots, is mixed. For example, a cross-sectional analysis in the predominantly White US Framingham Cohort Study observed an inverse association between serum 25-OHD with standard total computed tomography–derived quantities of subcutaneous adipose tissue and visceral adipose tissue; however, the investigators did not report similar inverse trends for 25-OHD with degree of insulin resistance or other obesity-related cardiometabolic risk factors, independent of adiposity.^30^ Other studies similarly did not report associations of total vitamin D status with obesity-related biomarkers independent of the measures of adiposity.^31^ In this present analysis, we did not observe further differences in response by WC. Thus, variability of total serum 25-OHD and 25-OHD3 levels with body weight appears to be associated with the amount of adiposity per se for some populations, rather than underlying cardiometabolic health state, although more research is needed in this area.

Parathyroid hormone levels correspond inversely to serum 25-OHD, with insufficient vitamin D leading to elevated PTH levels and normalization of PTH levels in response to supplementation.^32^ The threshold at which vitamin D–induced decreases in PTH levels plateau is hypothesized to reflect the optimal physiologic concentration of 25-OHD, and this association may differ by obesity status.^14^ Baseline PTH levels were higher in VITAL participants with higher BMIs; however, changes in PTH levels with vitamin D supplementation vs placebo did not differ significantly by BMI categories at 2 years’ follow-up. The overall minimal effect on PTH levels is consistent with the study population being predominantly vitamin D sufficient at baseline. Finally, the vitamin D carrier proteins, VDBP and albumin, were largely unchanged for all BMI categories, despite increases in total 25-OHD levels with supplementation.

### Limitations

This study has limitations. Our analysis of vitamin D supplementation and biomarkers of availability and activity of vitamin D was conducted in a large, US-based randomized clinical trial using vitamin D_3_, 2000 IU/d, vs placebo, with repeated blood sample collections and long-term follow-up. The cross-sectional analyses at baseline enrollment were adjusted for potential correlates of BMI and determinants of vitamin D status, including age, sex, race and ethnicity, season of blood draw, physical activity, and more. Residual confounding, however, may in part explain the differences by baseline BMI. The prospective analysis of vitamin D supplementation vs placebo with 2-year changes in vitamin D–related biomarkers benefits from randomized treatment assignment. Differences in postrandomization factors related to BMI, such as adherence to study pills or loss to follow-up, may bias our findings; however, dropout among the subset with repeated blood sample collection was minimal, and 94% were adherent to taking study pills. It is plausible that factors related to obesity explain the heterogeneity we observed by BMI category; reassurance is provided by estimates from multivariable-adjusted models.

## Conclusions

In this cohort study providing an explanatory analysis of a large randomized clinical trial, vitamin D_3_, 2000 IU/d, increased total 25-OHD levels as well as novel markers of vitamin D status, including 25-OHD3, FVD, and BioD, vs placebo at 2 years of intervention. Body mass index status modified the result of supplementation, with lower response and achieved levels for these biomarkers at higher BMIs. Vitamin D–binding protein and albumin levels were unchanged with supplementation and reductions in PTH levels with increased circulating vitamin D levels were consistent across BMI categories. Sequestering of vitamin D and its metabolites from the circulation into adipose tissue may contribute to lower serum concentrations and may at least, in part, explain the reduced effectiveness of supplementation previously reported for cancer end points among VITAL participants with obesity. Obesity-related pathophysiologic factors, such as impaired vitamin D receptor sensitivity, are not ruled out and further mechanistic research is warranted. The effective dose to achieve optimal circulating and bioactive concentrations required for prevention of cancer and diabetes or other benefits may therefore be higher among patients with excess adiposity, and nutri-kinetics will elucidate this further.

## Notes

Supplement 1. **eMethods.** Calculation of Bioactive Vitamin D (BioD) **eTable 1.** Characteristics of VITAL Participants Included in Analyses of Treatment Effect at 2 Years **eTable 2.** Multivariable-Adjusted Mean (Standard Error) or Geometric Mean (95% CI) Vitamin D–Related Biomarkers at Baseline and 2 Years Follow-up, by Randomized Treatment Assignment and Baseline WC (cm) **eTable 3.** Multivariable-Adjusted Mean (Standard Error) or Geometric Mean (95% CI) Vitamin D–Related Biomarkers at Baseline and 2 Years Follow-up, by Randomized Treatment Assignment and Baseline Body Mass Index Restricted to Low Serum Vitamin D (<20.0 ng/mL) at Baseline Randomization **eFigure.** Waterfall Plots of Individual Participant Response, as Change in Serum Total 25-OHD Concentrations (ng/mL), for (A) Placebo and (B) Vitamin D Supplementation at 2 Years Follow-up

Supplement 2.
Data Sharing Statement

## References

[1] Yin L, Ordóñez-Mena JM, Chen T, Schöttker B, Arndt V, Brenner H. Circulating 25-hydroxyvitamin D serum concentration and total cancer incidence and mortality: a systematic review and meta-analysis. Prev Med. 2013;57(6):753-764. doi:10.1016/j.ypmed.2013.08.026 24036014

[2] Zhang R, Li B, Gao X, . Serum 25-hydroxyvitamin D and the risk of cardiovascular disease: dose-response meta-analysis of prospective studies. Am J Clin Nutr. 2017;105(4):810-819. doi:10.3945/ajcn.116.140392 28251933

[3] Manson JE, Cook NR, Lee IM, ; VITAL Research Group. Vitamin D supplements and prevention of cancer and cardiovascular disease. N Engl J Med. 2019;380(1):33-44. doi:10.1056/NEJMoa1809944 30415629 PMC6425757

[4] Keum N, Lee DH, Greenwood DC, Manson JE, Giovannucci E. Vitamin D supplementation and total cancer incidence and mortality: a meta-analysis of randomized controlled trials. Ann Oncol. 2019;30(5):733-743. doi:10.1093/annonc/mdz059 30796437 PMC6821324

[5] Barbarawi M, Kheiri B, Zayed Y, . Vitamin D supplementation and cardiovascular disease risks in more than 83 000 individuals in 21 randomized clinical trials: a meta-analysis. JAMA Cardiol. 2019;4(8):765-776. doi:10.1001/jamacardio.2019.1870 31215980 PMC6584896

[6] Chandler PD, Chen WY, Ajala ON, ; VITAL Research Group. Effect of vitamin D_3_ supplements on development of advanced cancer: a secondary analysis of the VITAL randomized clinical trial. JAMA Netw Open. 2020;3(11):e2025850. doi:10.1001/jamanetworkopen.2020.25850 33206192 PMC7675103

[7] Hahn J, Cook NR, Alexander EK, . Vitamin D and marine omega 3 fatty acid supplementation and incident autoimmune disease: VITAL randomized controlled trial. BMJ. 2022;376:e066452. doi:10.1136/bmj-2021-066452 35082139 PMC8791065

[8] Barbarawi M, Zayed Y, Barbarawi O, . Effect of vitamin D supplementation on the incidence of diabetes mellitus. J Clin Endocrinol Metab. 2020;105(8):dgaa335. doi:10.1210/clinem/dgaa335 32491181

[9] Zhang Y, Tan H, Tang J, . Effects of vitamin D supplementation on prevention of type 2 diabetes in patients with prediabetes: a systematic review and meta-analysis. Diabetes Care. 2020;43(7):1650-1658. doi:10.2337/dc19-1708 33534730

[10] Ekwaru JP, Zwicker JD, Holick MF, Giovannucci E, Veugelers PJ. The importance of body weight for the dose response relationship of oral vitamin D supplementation and serum 25-hydroxyvitamin D in healthy volunteers. PLoS One. 2014;9(11):e111265. doi:10.1371/journal.pone.0111265 25372709 PMC4220998

[11] Vimaleswaran KS, Berry DJ, Lu C, ; Genetic Investigation of Anthropometric Traits-GIANT Consortium. Causal relationship between obesity and vitamin D status: bi-directional mendelian randomization analysis of multiple cohorts. PLoS Med. 2013;10(2):e1001383. doi:10.1371/journal.pmed.1001383 23393431 PMC3564800

[12] Bassatne A, Chakhtoura M, Saad R, Fuleihan GEH. Vitamin D supplementation in obesity and during weight loss: a review of randomized controlled trials. Metabolism. 2019;92:193-205. doi:10.1016/j.metabol.2018.12.010 30615949

[13] Chou SH, Murata EM, Yu C, . Effects of vitamin D_3_ supplementation on body composition in the Vitamin D and Omega-3 Trial (VITAL). J Clin Endocrinol Metab. 2021;106(5):1377-1388. doi:10.1210/clinem/dgaa981 33513226 PMC8063236

[14] Shapses SA, Lee EJ, Sukumar D, Durazo-Arvizu R, Schneider SH. The effect of obesity on the relationship between serum parathyroid hormone and 25-hydroxyvitamin D in women. J Clin Endocrinol Metab. 2013;98(5):E886-E890. doi:10.1210/jc.2012-3369 23509103 PMC3644609

[15] Drincic AT, Armas LAG, Van Diest EE, Heaney RP. Volumetric dilution, rather than sequestration best explains the low vitamin D status of obesity. Obesity (Silver Spring). 2012;20(7):1444-1448. doi:10.1038/oby.2011.404 22262154

[16] Roizen JD, Long C, Casella A, . Obesity decreases hepatic 25-hydroxylase activity causing low serum 25-hydroxyvitamin D. J Bone Miner Res. 2019;34(6):1068-1073. doi:10.1002/jbmr.3686 30790351 PMC6663580

[17] Pittas AG, Dawson-Hughes B, Sheehan P, ; D2d Research Group. Vitamin D supplementation and prevention of type 2 diabetes. N Engl J Med. 2019;381(6):520-530. doi:10.1056/NEJMoa1900906 31173679 PMC6993875

[18] Manson JE, Bassuk SS, Lee IM, . The Vitamin D and Omega-3 Trial (VITAL): rationale and design of a large randomized controlled trial of vitamin D and marine omega-3 fatty acid supplements for the primary prevention of cancer and cardiovascular disease. Contemp Clin Trials. 2012;33(1):159-171. doi:10.1016/j.cct.2011.09.009 21986389 PMC3253961

[19] Bassuk SS, Manson JE, Lee IM, . Baseline characteristics of participants in the Vitamin D and Omega-3 Trial (VITAL). Contemp Clin Trials. 2016;47:235-243. doi:10.1016/j.cct.2015.12.022 26767629 PMC4818165

[20] Powe CE, Evans MK, Wenger J, . Vitamin D–binding protein and vitamin D status of Black Americans and White Americans. N Engl J Med. 2013;369(21):1991-2000. doi:10.1056/NEJMoa1306357 24256378 PMC4030388

[21] Binkley N, Carter GD. Toward clarity in clinical vitamin D status assessment: 25(OH)D assay standardization. Endocrinol Metab Clin North Am. 2017;46(4):885-899. doi:10.1016/j.ecl.2017.07.012 29080641

[22] Tennant PWG, Arnold KF, Ellison GTH, Gilthorpe MS. Analyses of ‘change scores’ do not estimate causal effects in observational data. Int J Epidemiol. 2022;51(5):1604-1615. doi:10.1093/ije/dyab050 34100077 PMC9557845

[23] Clifton L, Clifton DA. The correlation between baseline score and post-intervention score, and its implications for statistical analysis. Trials. 2019;20(1):43. doi:10.1186/s13063-018-3108-3 30635021 PMC6330413

[24] Luttmann-Gibson H, Mora S, Camargo CA, . Serum 25-hydroxyvitamin D in the Vitamin D and Omega-3 Trial (VITAL): clinical and demographic characteristics associated with baseline and change with randomized vitamin D treatment. Contemp Clin Trials. 2019;87:105854. doi:10.1016/j.cct.2019.105854 31669447 PMC6875603

[25] Wortsman J, Matsuoka LY, Chen TC, Lu Z, Holick MF. Decreased bioavailability of vitamin D in obesity. Am J Clin Nutr. 2000;72(3):690-693. doi:10.1093/ajcn/72.3.690 10966885

[26] Martini LA, Wood RJ. Vitamin D status and the metabolic syndrome. Nutr Rev. 2006;64(11):479-486. doi:10.1111/j.1753-4887.2006.tb00180.x 17131943

[27] Mason C, Xiao L, Imayama I, . Effects of weight loss on serum vitamin D in postmenopausal women. Am J Clin Nutr. 2011;94(1):95-103. doi:10.3945/ajcn.111.015552 21613554 PMC3127511

[28] Aldenbäck E, Johansson HE. Anthropometric measurements and correlations to glucometabolic and cardiovascular risk in obese patients undergoing gastric bypass surgery. J Obes. 2021;2021:6647328. doi:10.1155/2021/6647328 34327018 PMC8310453

[29] Elkhwanky MS, Kummu O, Piltonen TT, . Obesity represses CYP2R1, the vitamin D 25-hydroxylase, in the liver and extrahepatic tissues. JBMR Plus. 2020;4(11):e10397. doi:10.1002/jbm4.10397 33210060 PMC7657391

[30] Cheng S, Massaro JM, Fox CS, . Adiposity, cardiometabolic risk, and vitamin D status: the Framingham Heart Study. Diabetes. 2010;59(1):242-248. doi:10.2337/db09-1011 19833894 PMC2797928

[31] Mousa A, Naderpoor N, de Courten MPJ, Scragg R, de Courten B. 25-Hydroxyvitamin D is associated with adiposity and cardiometabolic risk factors in a predominantly vitamin D–deficient and overweight/obese but otherwise healthy cohort. J Steroid Biochem Mol Biol. 2017;173:258-264. doi:10.1016/j.jsbmb.2016.12.008 28007531

[32] Saliba W, Barnett O, Rennert HS, Lavi I, Rennert G. The relationship between serum 25(OH)D and parathyroid hormone levels. Am J Med. 2011;124(12):1165-1170. doi:10.1016/j.amjmed.2011.07.009 22114830

