# Supplementary material for: Association of Body Weight With Response to Vitamin D Supplementation and Metabolism
Source: JAMA Netw Open. 2023 Jan 17;6(1):e2250681. doi: 10.1001/jamanetworkopen.2022.50681 (PMC9856931; doi:10.1001/jamanetworkopen.2022.50681)
Supplement: Supplement 1. — eMethods. Calculation of Bioactive Vitamin D (BioD) eTable 1. Characteristics of VITAL Participants Included in Analyses of Treatment Effect at 2 Years eTable 2. Multivariable-Adjusted Mean (Standard Error) or Geometric Mean (95% CI) Vitamin D–Related Biomarkers at Baseline and 2 Years Follow-up, by Randomized Treatment Assignment and Baseline WC (cm) eTable 3. Multivariable-Adjusted Mean (Standard Error) or Geometric Mean (95% CI) Vitamin D–Related Biomarkers at Baseline and 2 Years Follow-up, by Randomized Treatment Assignment and Baseline Body Mass Index Restricted to Low Serum Vitamin D (<20.0 ng/mL) at Baseline Randomization eFigure. Waterfall Plots of Individual Participant Response, as Change in Serum Total 25-OHD Concentrations (ng/mL), for (A) Placebo and (B) Vitamin D Supplementation at 2 Years Follow-up [file jamanetwopen-e2250681-s001.pdf]

Supplementary Online Content

Tobias DK, Luttmann-Gibson H, Mora S, et al. Association of body weight with response to vitamin D supplementation and metabolism. *JAMA Netw Open*. 2023;6(1):e2250681. doi:10.1001/jamanetworkopen.2022.50681

eMethods. Calculation of Bioactive Vitamin D (BioD)

eTable 1. Characteristics of VITAL Participants Included in Analyses of Treatment Effect at 2 Years

eTable 2. Multivariable-Adjusted Mean (Standard Error) or Geometric Mean (95% CI) Vitamin D–Related Biomarkers at Baseline and 2 Years Follow-up, by Randomized Treatment Assignment and Baseline WC (cm)

eTable 3. Multivariable-Adjusted Mean (Standard Error) or Geometric Mean (95% CI) Vitamin D–Related Biomarkers at Baseline and 2 Years Follow-up, by Randomized Treatment Assignment and Baseline Body Mass Index Restricted to Low Serum Vitamin D (<20.0 ng/mL) at Baseline Randomization

eFigure. Waterfall Plots of Individual Participant Response, as Change in Serum Total 25-OHD Concentrations (ng/mL), for (A) Placebo and (B) Vitamin D Supplementation at 2 Years Follow-up

This supplementary material has been provided by the authors to give readers additional information about their work.

© 2023 Tobias DK et al. *JAMA Network Open.*

eMethods

Calculation of bioactive vitamin D (BioD)

BioD values in VITAL were calculated based on the equation by Powe et al (Powe C, et al, NEJM 2013). Briefly, their calculation estimates the amount of 25-OHD that is unbound to VDBP, and thus either free or bound to albumin in circulation. The equation applies the affinity constants of 25-OHD with VDBP of $K_{VDBP}=0.7 \times 10^{9}$ M^{-1}, and 25-OHD with albumin of $K_{ALB}=6 \times 10^{5}$ M^{-1}.

Thus, with the measured albumin and free 25-OHD (FVD), we calculated the following:

$$BioD = (K_{ALB} * ALBUMIN + 1) * FVD$$

© 2023 Tobias DK et al. *JAMA Network Open.*

eTable 1. Characteristics of VITAL participants included in analyses of treatment effect at 2 years.

	BMI (kg/m^2) at baseline randomization				
	**Underweight <18.5**	**Normal 18.5-24.9**	**Overweight 25.0-29.9**	**Obesity I 30.0-34.9**	**Obesity II ≥35.0**
N	26	724	1072	547	373
BMI, kg/m^2 – mean (SD)	17.4 (1.1)	22.8 (1.5)	27.3 (1.4)	32.2 (1.4)	39.9 (4.5)
Age, years – mean (SD)	67.4 (7.5)	67.4 (6.7)	66.6 (6.9)	66.3 (6.4)	64.8 (6.2)
Female – %	76.9	52.1	38.4	49.0	57.1
Race or ethnic group – %					
Non-Hispanic white	83.3	80.6	76.4	69.5	60.7
Black	4.2	9.1	15.9	22.1	31.3
Nonblack Hispanic	0.0	2.7	4.1	4.7	5.2
Asian or Pacific Islander	12.5	6.0	1.0	0.6	0.3
Native American, Alaskan Native	0.0	0.3	0.8	1.3	1.1
Other or unknown	0.0	1.3	1.8	1.9	1.4
Highest education – %					
No high school	4.0	1.0	0.5	1.3	3.2
High school	4.0	4.7	8.9	11.9	16.4
College	40.0	38.2	38.5	45.6	49.6
Post college	52.0	56.1	52.1	41.2	30.8
Exercise, MET-hrs/wk – mean (SD)	33.8 (32.9)	29.4 (27.1)	25.6 (24.8)	17.9 (20.5)	12.6 (24.0)
Smoking – %					
Never	61.5	55.4	47.2	45.6	49.7
Past	34.6	38.0	47.3	49.4	41.6
Current	3.8	6.7	5.5	5.0	8.6
Alcohol intake – %					
Never	38.5	23.1	25.3	35.3	52.5
<1/week	7.7	6.9	7.0	8.2	7.1
1-6/week	42.3	36.7	39.1	35.3	28.0
Daily	11.5	33.4	28.6	21.2	12.4
Diabetes – %	19.2	22.0	36.2	54.3	67.0
Hypertension medication – %	23.1	39.9	54.9	70.4	85.3
Cholesterol-lowering medication – %	23.1	34.8	48.1	58.0	62.2

© 2023 Tobias DK et al. *JAMA Network Open.*

Pre-trial supplemental vitamin D – %	34.6	49.2	55.5	58.3	57.4
Geographic region – %					
West	15.4	12.4	10.1	12.1	9.1
South	30.8	18.1	24.3	29.6	35.4
Midwest	7.7	15.2	17.7	21.9	27.3
Northeast	46.2	54.3	47.9	36.4	28.2
Season at blood draw – %					
Winter	11.5	22.2	23.9	22.9	27.1
Spring	23.1	22.0	21.1	21.9	20.9
Summer	42.3	23.5	23.2	20.1	23.1
Fall	23.1	32.3	31.8	35.1	29.0
CTSC sub-study participation – %	46.2	45.7	38.5	25.2	18.8

SD=standard deviation; BMI=body mass index; MET=metabolic equivalent of tasks; CTSC=Clinical and Translational Science Center

© 2023 Tobias DK et al. *JAMA Network Open.*

eTable 2. Multivariable-adjusted mean (standard error) or geometric mean (95% CI) vitamin D-related biomarkers at baseline and 2 years follow-up, by randomized treatment assignment and baseline WC (cm).

	**Placebo**		**Vitamin D**			
	**Baseline**	**Year 2**	**Baseline**	**Year 2**		
**Biomarker^**<br>WC category	**Multivariable-Adjusted Mean (SEM) or Geometric Mean (95% CI)**		**Multivariable-Adjusted Mean (SEM) or Geometric Mean (95% CI)**		**Treatment Effect Mean (SE)**	**P-value Treatment Effect Interaction by WC**
**Total 25-OHD – ng/mL**						
I – Minimal risk	29.5 (0.7)	29.4 (0.8)	29.2 (0.7)	42.5 (0.7)	13.1 (0.9)	0.001
II – Moderate risk	29.1 (0.7)	29.4 (0.8)	28.3 (0.7)	41.2 (0.8)	12.3 (1.0)	
III – High risk	28.3 (0.5)	27.7 (0.5)	26.5 (0.5)	37.9 (0.6)	11.6 (0.6)	
**25-OHD3 – ng/mL**						
I – Minimal risk	29.2 (0.7)	29.0 (0.8)	28.9 (0.7)	42.4 (0.7)	13.4 (0.9)	0.003
II – Moderate risk	28.7 (0.7)	29.2 (0.8)	27.9 (0.7)	41.1 (0.8)	12.4 (1.0)	
III – High risk	28.0 (0.5)	27.0 (0.5)	26.0 (0.5)	37.8 (0.6)	12.5 (0.6)	
**Free vitamin D – pg/mL**						
I – Minimal risk	6.39 (0.16)	6.23 (0.18)	6.50 (0.16)	10.20 (0.18)	3.84 (0.21)	<0.001
II – Moderate risk	6.11 (0.15)	6.42 (0.19)	6.02 (0.15)	9.59 (0.19)	3.15 (0.23)	
III – High risk	5.81 (0.10)	5.81 (0.12)	5.65 (0.10)	8.37 (0.13)	2.76 (0.14)	
**Bioavailable vitamin D – ng/mL**						
I – Minimal risk	2.4 (2.2, 2.5)	2.3 (2.2, 2.4)	2.4 (2.3, 2.5)	3.9 (3.7, 4.1)	1.5 (0.1)	<0.001
II – Moderate risk	2.3 (2.2, 2.4)	2.4 (2.3, 2.5)	2.2 (2.1, 2.4)	3.6 (3.4, 3.8)	1.2 (0.1)	
III – High risk	2.2 (2.1, 2.2)	2.1 (2.0, 2.2)	2.1 (2.0, 2.2)	3.2 (3.0, 3.3)	1.1 (0.1)	
**VDBP – mg/mL**						
I – Minimal risk	452 (441, 463)	451 (440, 462)	448 (438, 459)	445 (435, 456)	-4 (6)	0.81
II – Moderate risk	451 (440, 462)	446 (435, 457)	450 (439, 461)	450 (440, 462)	8 (5)	

© 2023 Tobias DK et al. *JAMA Network Open.*

III – High risk	457 (449, 465)	451 (443, 459)	447 (438, 455)	448 (440, 457)	4 (4)	
**Albumin – g/dL**						
I – Minimal risk	4.31 (0.02)	4.31 (0.02)	4.32 (0.02	4.31 (0.02)	0.001 (0.03)	0.15
II – Moderate risk	4.36 (0.02)	4.32 (0.02)	4.33 (0.02)	4.30 (0.02)	0.01 (0.03)	
III – High risk	4.35 (0.01)	4.33 (0.01)	4.30 (0.01)	4.32 (0.02)	0.01 (0.02)	
**PTH – pg/mL**						
I – Minimal risk	34.1 (32.0, 36.3)	34.7 (32.7, 36.9)	35.5 (33.4, 37.8)	33.5 (31.6, 35.6)	-2.4 (1.3)	0.45
II – Moderate risk	36.8 (34.4, 39.2)	35.4 (33.2, 37.7)	35.3 (33.0, 37.7)	32.1 (30.1, 34.2)	-1.3 (1.3)	
III – High risk	38.4 (36.6, 40.2)	37.8 (36.0, 39.6)	42.1 (40.1, 44.3)	38.6 (36.7, 40.6)	-1.7 (1.1)	
**Calcium – mg/dL**						
I – Minimal risk	9.20 (9.14, 9.26)	9.14 (9.08, 9.20)	9.20 (9.15, 9.26)	9.16 (9.11, 9.22)	0.03 (0.04)	0.17
II – Moderate risk	9.24 (9.18, 9.30)	9.20 (9.15, 9.26)	9.20 (9.14, 9.26)	9.18 (9.13, 9.24)	0.00 (0.04)	
III – High risk	9.31 (9.27, 9.35)	9.27 (9.23, 9.31)	9.25 (9.21, 9.29)	9.26 (9.21, 9.30)	0.02 (0.03)	

WHO cut-points for waist circumference (WC; cm) – I: women <80.0, men <94.0; II: women 80.0-87.9, men 94.0-101.9; III: women 88.0+, men 102.0+. N=number of participants, VDBP=vitamin D binding protein, PTH=parathyroid hormone

Values are least square mean (standard error of the mean [SEM]) or ^geometric mean (95% CI [confidence interval]) adjusted for baseline factors: age (continuous), sex (male, female), prevalence of type 2 diabetes, pre-intervention vitamin D supplement use, smoking status (never, past, current, not reported), total physical activity (above, below median=16.6 MET-hrs/week), race/ethnicity (non-Hispanic White, Black, non-black Hispanic, Asian or Pacific Islander, Native American or Alaskan Native, other/unknown, not reported), geographic region (West, South, Midwest, Northeast), season of blood draw (Winter, Spring, Summer, Fall), and in-person CTSC subgroup participation. Average treatment effects are additionally adjusted for baseline biomarker concentrations. Tests for interaction of treatment effect by baseline WC category are additionally adjusted for baseline biomarker concentrations and baseline biomarker*WC interaction.

^Sample size included for each biomarker: total 25-OHD n=961, 25-OHD3 n=991, free vitamin D n=961, bioavailable vitamin D n=960, vitamin D binding protein n=961, albumin n=960, PTH n=955, calcium n=960

© 2023 Tobias DK et al. *JAMA Network Open.*

eTable 3. Multivariable-adjusted mean (standard error) or geometric mean (95% CI) vitamin D-related biomarkers at baseline and 2 years follow-up, by randomized treatment assignment and baseline body mass index– restricted to low serum vitamin D (<20.0 ng/mL) at baseline randomization.

	Placebo		Vitamin D			
	Baseline	Year 2	Baseline	Year 2		
**Biomarker^**<br>BMI category – kg/m^2	**Multivariable-Adjusted Mean (SEM) or Geometric Mean (95% CI)**		**Multivariable-Adjusted Mean (SEM) or Geometric Mean (95% CI)**		**Treatment Effect**	**P-value Interaction by Baseline BMI**
**Total 25-OHD – ng/mL**						
<25.0	15.6 (0.4)	20.9 (1.4)	15.0 (0.4)	35.2 (1.5)	15.8 (2.2)	0.085
25.0-29.9	15.4 (0.3)	19.8 (1.1)	15.0 (0.3)	36.3 (1.0)	17.5 (1.7)	
30.0-34.9	15.1 (0.5)	21.0 (1.4)	14.7 (0.6)	32.0 (1.5)	11.2 (2.8)	
≥35.0	14.9 (0.5)	18.7 (1.7)	13.3 (0.4)	31.9 (1.5)	14.3 (3.1)	
**25-OHD3 – ng/mL**						
<25.0	15.5 (0.4)	19.8 (1.4)	14.9 (0.4)	35.1 (1.4)	16.4 (2.2)	0.016
25.0-29.9	15.3 (0.3)	18.3 (1.0)	15.1 (0.3)	36.3 (0.9)	19.5 (1.5)	
30.0-34.9	15.0 (0.5)	19.0 (1.3)	14.5 (0.6)	31.9 (1.4)	12.4 (2.7)	
≥35.0	14.5 (0.5)	17.3 (1.7)	13.0 (0.4)	31.7 (1.4)	15.2 (2.9)	
**Free vitamin D – pg/mL**						
<25.0	3.50 (0.12)	4.14 (0.42)	3.61 (0.13)	8.77 (0.45)	5.55 (0.75)	0.002
25.0-29.9	3.48 (0.11)	4.41 (0.37)	3.41 (0.09)	7.64 (0.33)	3.81 (0.61)	
30.0-34.9	3.88 (0.13)	4.75 (0.31)	2.16 (0.21)	5.42 (0.47)	-9.26 (12.78)	
≥35.0	---	---	---	---	---	
**Bioavailable vitamin D – ng/mL**						
<25.0	1.3 (1.2, 1.4)	1.6 (1.4, 1.8)	1.4 (1.3, 1.5)	3.1 (2.7, 3.6)	2.1 (0.3)	0.003
25.0-29.9	1.4 (1.3, 1.5)	1.6 (1.4, 1.8)	1.3 (1.2, 1.4)	2.9 (2.6, 3.2)	1.6 (0.3)	
30.0-34.9	1.8 (1.6, 2.1)	2.2 (1.9, 2.4)	0.6 (0.5, 0.7)	1.2 (1.1, 1.5)	-3.4 (4.1)	
≥35.0	---	---	---	---	---	
**VDBP – mg/mL**						

© 2023 Tobias DK et al. *JAMA Network Open.*

<25.0	451 (432, 471)	449 (426, 474)	434 (414, 456)	443 (418, 471)	12 (16)	0.35
25.0-29.9	443 (425, 463)	444 (423, 467)	430 (414, 447)	442 (423, 461)	6 (17)	
30.0-34.9	384 (350, 422)	374 (357, 392)	544 (476, 621)	492 (458, 529)	60 (96)	
≥35.0	---	---	---	---	---	
**Albumin – g/dL**						
<25.0	4.38 (0.03)	4.40 (0.04)	4.37 (0.03)	4.34 (0.04)	-0.07 (0.05)	0.83
25.0-29.9	4.44 (0.03)	4.40 (0.03)	4.39 (0.02)	4.38 (0.03)	0.02 (0.04)	
30.0-34.9	4.33 (0.05)	4.34 (0.04)	4.32 (0.05)	4.24 (0.04)	-0.08 (0.05)	
≥35.0	4.17 (0.04)	4.16 (0.04)	4.21 (0.03)	4.19 (0.03)	-0.04 (0.05)	
**PTH – pg/mL**						
<25.0	42.8 (38.4, 47.7)	41.1 (36.7, 45.8)	49.0 (43.8, 54.7)	40.5 (36.1, 45.4)	-7.2 (4.0)	0.95
25.0-29.9	43.2 (39.4, 47.3)	41.1 (337.4, 45.1)	45.9 (42.2, 49.9)	37.1 (34.1, 40.4)	-8.4 (2.5)	
30.0-34.9	51.3 (45.3, 58.2)	43.0 (37.9, 48.7)	47.6 (41.3, 54.7)	42.1 (36.6, 48.4)	-1.7 (5.5)	
≥35.0	48.1 (41.7, 55.5)	50.0 (44.4, 56.2)	55.6 (49.5, 62.5)	47.0 (42.7, 51.7)	-8.2 (4.5)	
**Calcium – mg/dL**						
<25.0	9.23 (9.11, 9.35)	9.32 (9.19, 9.44)	9.26 (9.15, 9.38)	9.16 (9.04, 9.28)	-0.19 (0.08)	0.50
25.0-29.9	9.33 (9.24, 9.41)	9.29 (9.21, 9.37)	9.30 (9.22, 9.38)	9.34 (9.27, 9.42)	0.06 (0.06)	
30.0-34.9	9.30 (9.19, 9.40)	9.36 (9.28, 9.44)	9.33 (9.20, 9.45)	9.24 (9.14, 9.34)	-0.24 (0.10)	
≥35.0	9.33 (9.20, 9.47)	9.28 (9.17, 9.39)	9.16 (9.06, 9.27)	9.25 (9.15, 9.35)	-0.03 (0.12)	

BMI=body mass index, N=number of participants, VDBP=vitamin D binding protein, PTH=parathyroid hormone

Values are least square mean (standard error of the mean [SEM]) or ^geometric mean (95% CI [confidence interval]) adjusted for baseline factors: age (continuous), sex (male, female), prevalence of type 2 diabetes, pre-intervention vitamin D supplement use, smoking status (never, past, current, not reported), total physical activity (above, below median=16.6 MET-hrs/week), race/ethnicity (non-Hispanic White, Black, non-black Hispanic, Asian or Pacific Islander, Native American or Alaskan Native, other/unknown, not reported), geographic region (West, South, Midwest, Northeast), season of blood draw (Winter, Spring, Summer, Fall), and in-person CTSC subgroup participation. Average treatment effects are

© 2023 Tobias DK et al. *JAMA Network Open.*

additionally adjusted for baseline biomarker concentrations. Tests for interaction of treatment effect by baseline BMI category are additionally adjusted for baseline biomarker concentrations and baseline biomarker*BMI interaction.

^Sample size included for each biomarker: total 25-OHD n=397, 25-OHD3 n=396, free vitamin D n=158, bioavailable vitamin D n=158, vitamin D binding protein n=158, albumin n=429, PTH n=427, calcium n=331

© 2023 Tobias DK et al. *JAMA Network Open.*

eFigure. Waterfall plots of individual participant response, as change in serum total 25-OHD concentrations (ng/mL), for (A) placebo and (B) vitamin D supplementation at 2 years follow-up.

(A) Change at 2 years among placebo.

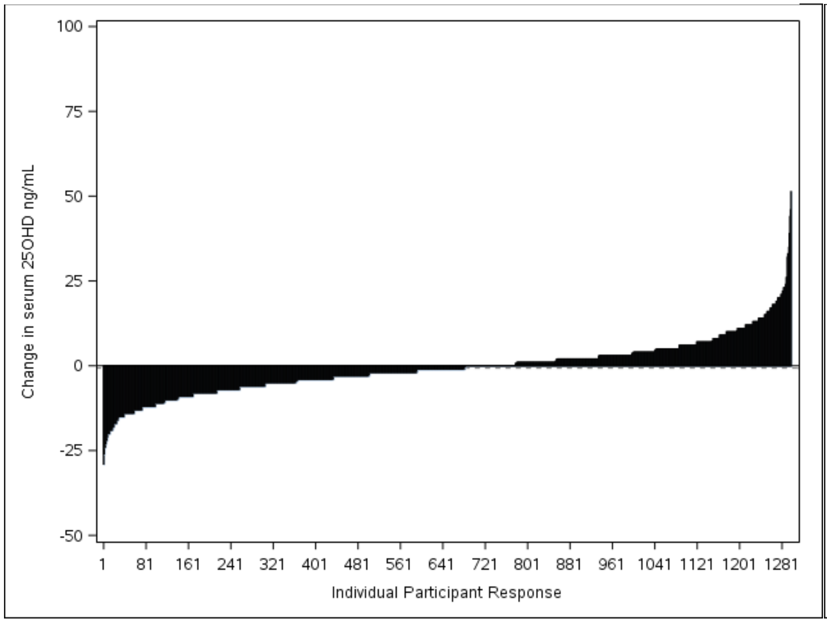

(B) Change at 2 years among active vitamin D supplementation.

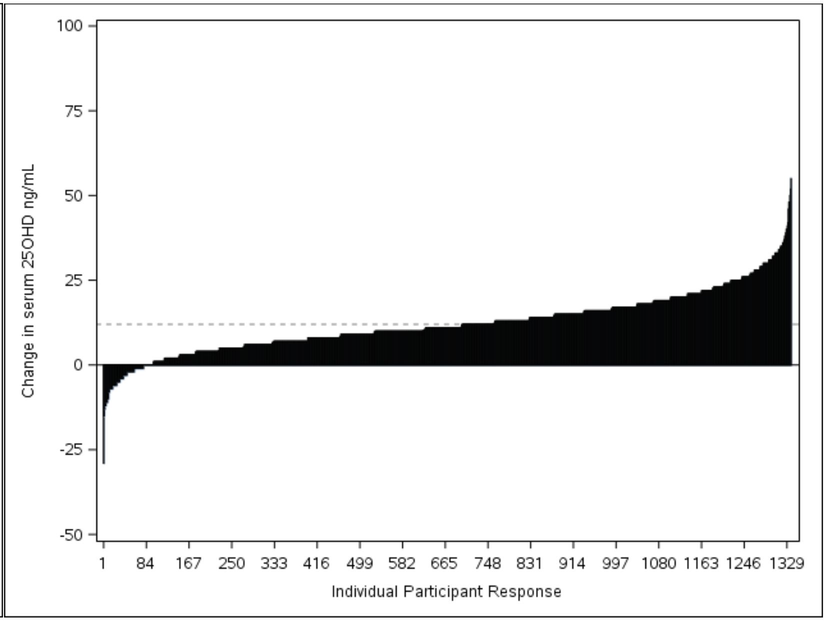

© 2023 Tobias DK et al. *JAMA Network Open.*
