# Supplementary material for: Association of Body Weight With Response to Vitamin D Supplementation and Metabolism
Source: JAMA Netw Open. 2023 Jan 17;6(1):e2250681. doi: 10.1001/jamanetworkopen.2022.50681 (PMC9856931; doi:10.1001/jamanetworkopen.2022.50681)
Supplement: Supplement 2. — Data Sharing Statement [file jamanetwopen-e2250681-s002.pdf]

Data Sharing Statement

Tobias. Association of Body Weight With Response to Vitamin D Supplementation and Metabolism. *JAMA Netw Open*. Published January 17, 2023.
doi:10.1001/jamanetworkopen.2022.50681

Data
Data available: Yes
Data types: Deidentified participant data
How to access data: Users of Project Datasphere need to apply to become an authorized user by filling out a form-- once approved, they'll have access to the data files there. Specific instructions can be found below: https://www.projectdatasphere.org/data-platform/access-data There's a link to the application form itself on that page but it can be also be found here: https://data.projectdatasphere.org/projectdatasphere/html/registration
When available: With publication

Supporting Documents
Document types: None

Additional Information
Who can access the data: Users of Project Datasphere need to apply to become an authorized user by filling out a form-- once approved, they'll have access to the data files there. Specific instructions can be found below: https://www.projectdatasphere.org/data-platform/access-data There's a link to the application form itself on that page but it can be also be found here: https://data.projectdatasphere.org/projectdatasphere/html/registration
Types of analyses: Users of Project Datasphere need to apply to become an authorized user by filling out a form-- once approved, they'll have access to the data files there. Specific instructions can be found below: https://www.projectdatasphere.org/data-platform/access-data There's a link to the application form itself on that page but it can be also be found here: https://data.projectdatasphere.org/projectdatasphere/html/registration
Mechanisms of data availability: Users of Project Datasphere need to apply to become an authorized user by filling out a form-- once approved, they'll have access to the data files there. Specific instructions can be found below: https://www.projectdatasphere.org/data-platform/access-data There's a link to the application form itself on that page but it can be also be found here: https://data.projectdatasphere.org/projectdatasphere/html/registration
